# Therapeutic Strategies for the Treatment of Chronic Hyperuricemia: An Evidence-Based Update

**DOI:** 10.3390/medicina57010058

**Published:** 2021-01-10

**Authors:** Arrigo F. G. Cicero, Federica Fogacci, Masanari Kuwabara, Claudio Borghi

**Affiliations:** 1 Hypertension Research Unit, Department of Medical and Surgical Sciences, University of Bologna, 40126 Bologna, Italy; arrigo.cicero@unibo.it (A.F.G.C.); federicafogacci@gmail.com (F.F.); 2 Cardiology Department and Intensive Care Unit, Toranomon Hospital, Tokyo 40138, Japan; kuwamasa728@gmail.com

**Keywords:** hyperuricemia, uric acid, uric acid-lowering drugs, xanthine oxidase inhibitors, uricosurics

## Abstract

This article aims to critically review the evidence on the available therapeutic strategies for the treatment of hyperuricemia. For this reason, several papers were reviewed. Xanthine oxidase inhibitors are the safest and most effective uric acid lowering drugs for the management of chronic hyperuricemia, while the efficacy of uricosuric agents is strongly modulated by pharmacogenetics. Emergent drugs (lesinurad, peglotidase) were found to be more effective for the acute management of refractory hyperuricemia, but their use is supported by a relatively small number of clinical trials so that further well-designed clinical research is needed to deepen their efficacy and safety profile.

## 1. Introduction

Uric acid [UA] is mostly produced in the intestine and liver, as the final product of purine catabolism. This metabolic pathway has been highly conserved during the evolutionary process in most living species, with some exceptions such as Dalmatian dogs and some birds [1]. During normal homeostasis, serum levels of UA [SUA] are kept lower than 7 mg/dL in men and 6 mg/dL in women [2], mainly thanks to a complex regulation process involving the renal transport systems. However, chronic hyperuricemia might depend on the overproduction of UA and/or a reduced UA renal excretion [3], even though new pathogenic mechanisms also focus on ABCG2 expression in the intestine and the gut microbiota [4]. A number of factors are involved in determining SUA levels, including age (i.e., the prevalence of hyperuricemia increased from the age of 60 years, reaching a plateau after the age of 70 years), sex, cell renewal factor, renal function, and exogenous factors (i.e., dietetic factors, e.g., fructose, purine, and alcohol intake) [5].

Even though very high SUA is a well-known risk factor for gout, SUA levels at the upper limits of normal were shown to increase the chance of developing cardiovascular disease, type 2 diabetes, and chronic kidney disease [CKD] in the general population [6], and the risk of developing metabolic syndrome in elderly [7]. Recent evidence coming from the large epidemiological URRAH study shows that SUA is associated with a significantly increased risk of heart failure (hazard ratio, 1.65 [95% confidence interval—CI, 1.28–2.11]), fatal heart failure (hazard ratio, 1.65 [95% CI, 1.28–2.11]), total mortality (hazard ratio, 1.53 [95% CI, 1.21–1.93]), and cardiovascular death (hazard ratio, 2.08 [95% CI, 1.15–2.97]; *p* < 0.001) [8,9]. The risk seems to increase with SUA levels lower than the ones associated with an increased risk of gout and even lower to the currently considered normal values [10].

Elevated serum UA levels has been associated with greater inflammatory status and greater risk of mortality also in patients with a history of cardiovascular disease and in particular after myocardial infarction [11,12].

Worldwide the percentage of adults with hyperuricemia has been increasing over the past decades and, lately, novel insights into the pathophysiology of this disorder has led to significant advancement in its management which uses traditional agents for mild-to-moderate disease and cutting-edge drugs for individuals with severe or refractory hyperuricemia [13]. Therefore, to recognize which patients mainly overproduce UA, which ones mainly under excrete it, and which ones suffer from both of these conditions is of fundamental importance in clinical practice. This initial differentiation could lead to a more appropriate use of the available UA lowering drugs based on their pharmacodynamics [14].

This review aimed to summarize the main clinical indications and the pharmacological profile of the most clinically tested UA lowering drugs.

## 2. Drugs Reducing the Generation of Uric Acid: The Xanthine Oxidase Inhibitors

Xanthine oxidase [XO] is the form of enzyme xanthine dehydrogenase responsible for converting hypoxanthine to UA in the purine metabolism pathway. During this process, there is the production of reactive oxygen species [ROS] [15]. When produced in excess, ROS reduces the synthesis of nitric oxide and lead to endothelial dysfunction [15]. A meta-analysis pooling data from 81 randomized clinical trials (RCTs) (N. 10684 included patients) showed that the xanthine oxidase inhibitors [XOIs] decreases the risk of total and serious cardiovascular events [Odds Ratio—OR = 0.60, *p* = 0.001 and OR = 0.64, *p* < 0.01 respectively] and onset/worsening hypertension [OR = 0.54, *p* = 0.002] in comparison with placebo. Moreover, a sub-analysis pooling data from 9 trials and 616 hyperuricemic subjects found that XOIs are more effective in secondary prevention, reducing the occurrence of major cardiovascular adverse events in those high-risk patients [Relative Risk—RR = 0.42, *p* < 0.01] [16]. Certainly, the potential benefits attributed to XOIs may rely on their antioxidant properties other than SUA reduction, by the inhibition of ROS production [17].

These agents are the first line in urate-lowering therapy for gout, being effective in most hyperuricemic patients with an acceptable tolerability profile [18].

### 2.1. Allopurinol

Allopurinol and its metabolite oxypurinol are respectively analogs of hypoxanthine and xanthine, and decrease UA formation by binding and inhibiting XO [19]. This drug can be administered orally or parenterally, to treat gout and prevent the recurrence of kidney stones.

Allopurinol treatment is the mainstay in the prophylaxis of hyperuricemia in patients receiving chemotherapy [20]. Moreover, it was associated with an improvement in flow-mediated dilation [21] and a slowdown in CKD evolution [22,23]. An ongoing clinical trial (ClinicalTrial.gov ID: NCT03865407) will clarify the effect of allopurinol treatment on renal function in pediatric CKD patients with high UA levels and establish whether it alters renal injury biomarkers.

After oral administration, allopurinol is quickly absorbed in the upper gastrointestinal tract. The peak plasma concentration is reached in ~30 min after ingestion, and the plasma half-life is 2–3 h [24].

The main active metabolite of allopurinol is oxypurinol, which is filtered and partially reabsorbed in the kidneys, having the same mechanism of action as allopurinol but a plasma half-life of the order of 14–30 h [24].

Allopurinol has a dose-dependent SUA-lowering effect and is usually administered at a daily dose of 100 mg to 600 mg/day for the treatment of chronic hyperuricemia [25]—the maximum daily dose can however reach 800–900 mg/day, by country and product-label [21]. Notwithstanding that a meta-analysis has lately showed that only lower doses of allopurinol (≤300 mg/day) can reduce the risk of cardiovascular events (*p* < 0.001) [26].

In general, patients are recommended to start on a low dose of allopurinol and then gradually increase it. This expedient allows to contain the risk of hypersensitivity syndrome [AHS] and provides a chance to prevent acute gout attacks immediately after starting the treatment [17]. Moreover, the risk of acute attacks of gout can be further prevented by the co-administration of an anti-inflammatory drug or low-dose colchicine [27].

The most common reported side effects related to allopurinol use include gastrointestinal distress and skin rash ranging in severity. In addition to adverse skin reactions, treatment-emergent adverse events include AHS (which is rare but also potentially fatal), hepatitis, interstitial nephritis, and eosinophilia. CKD patients treated with thiazides have an increased risk for developing AHS so that patients with renal impairment are recommended to use lower allopurinol doses. Furthermore, pharmacogenomics plays a role in the safety of allopurinol use and the risk of serious adverse events augments in patients with the HLA-B*5801 haplotype, highly prevalent in Thai and Han Chinese ethnicities with at least stage 3 CKD [28].

Finally, allopurinol is contraindicated in patients on didanosine [29], while the concomitant administration of 300–600 mg/day allopurinol and azathioprine or mercaptopurine requires a reduction in dosage of the immunosuppressants to approximately one-third or one-fourth of the usual dose. In these cases, the therapeutic response and toxicity need to be monitored [30].

However, it must be acknowledged that these concerns can lead to allopurinol underdosing, and determining inadequate management of hyperuricemia of consequence [31].

### 2.2. Febuxostat

Febuxostat is an oral non-purine selective XO inhibitor able to blind and inhibit both the reduced and the oxidized form of XO. Febuxostat prevents enzyme turnover and blocks the active pterin–containing molybdenum center in enzyme-substrate complex, reducing the consequent ROS production [32]. Following oral administration, it is absorbed in the upper gastrointestinal tract and reaches the peak plasma concentration within 1 h. Its plasma half-life is of the order of 5–8 h.

Febuxostat is metabolized and excreted mainly through hepatic conjugation. It is more effective than allopurinol in reducing SUA concentrations and exerts also anti-inflammatory properties on the endothelium [33,34].

Febuxostat efficacy has been partly related to the inhibition of glycosaminoglycan-bound and endothelial XO cell-bound [35]. By comparing the effect of febuxostat and allopurinol in gouty patients, febuxostat is more effective in preventing arterial stiffening over 1 year [36]. Contrarily, a recent phase 4 randomized, double-blind, crossover designed, a placebo-controlled clinical trial showed that febuxostat does not significantly improve coronary endothelial dysfunction in patients with known stable cardiovascular artery disease, even though it lowers SUA [37].

The recommended daily dosage of febuxostat is 80–120 mg/day, even if remarkable reductions in SUA levels can be achieved also with lower dosages (i.e., 40 mg/day). In particular, treatment with 40 mg/day febuxostat seems to be able to reach the target value of SUA (<6 mg/dL) more easily than allopurinol 300 mg.

Febuxostat is indicated in hyperuricemic patients with gout, being a valid alternative treatment for individuals who experienced an allergic reaction with allopurinol [38]. No dosage adjustments are needed in patients with mild-to-moderate or severe hepatic impairment, though the American College of Rheumatology (ACR) recommends using low dose febuxostat (40 mg/die) in patients with severe renal impairment (eGFR < 30 mL/min/m^2^) [39]. The ongoing LUMINA trial (ClinicalTrial.gov ID: NCT03200210) is investigating the long-term effect of the drug on cardiovascular outcomes in continuous ambulatory peritoneal dialysis patients with hyperuricemia.

Treatment-emergent adverse events associated with febuxostat include stomach pain, diarrhea, muscle pain, and slight elevations in transaminases [40]. These observations have been corroborated by a recent meta-analysis that, pooling data from 13 RCTs (13,539 patients overall), concluded that febuxostat has a similar cardiovascular safety when compared with allopurinol (side effects OR = 0.72, *p* = 0.55) and a halved risk of skin reactions (OR = 0.50, *p* = 0.01) [41].

Recently febuxostat has been shown to be better tolerated than allopurinol in hyperuricemic and gouty patients, especially when renal failure was present [adverse events OR = 0.85] [42]. Febuxostat has also been investigated for its nephroprotective activity in comparison with allopurinol and has recently been shown to safely reduce SUA in patients after kidney transplantation [43,44].

For these reasons, even if the 2016 guidelines of the European League Against Rheumatism [EULAR] suggest the use of febuxostat as an alternative SUA lowering agent [45], the latest American College of Physicians’ guidelines (ACP 2017) suggest febuxostat as a first-line agent for the management of hyperuricemia [46]. The latest recommendation differs from the most recent one of the ACR placing allopurinol as the preferred first-line urate-lowering therapy, following the potential cardiovascular safety concerns attributed to febuxostat [39].

The Cardiovascular Safety of Febuxostat and Allopurinol in Patients with Gout and Cardiovascular Morbidities (CARES) trial reported a higher risk of cardiovascular mortality with the use of febuxostat versus control [47]. Following these results, the Food and Drug Administration (FDA) added a new boxed warning indicating that there is an increased risk of heart-related death and death from all causes with febuxostat. However, the available evidence regarding the effects of febuxostat on mortality and major cardiovascular adverse events in patients with gout is controversial. Most recently the Febuxostat versus Allopurinol Streamlined Trial (FAST) has yielded findings coming out in the opposite direction of CARES [48].

### 2.3. Topiroxostat

Topiroxostat is a selective XOI that has good oral bioavailability. Its pharmacologically active metabolite—namely the N-glucuronide topiroxostat (F11741)—is produced by the liver [49].

In vivo, topiroxostat causes a dose-dependent decrease in the excretion of urinary albumin [UAE] and the plasma activity of XO [50]. Then, similar findings were safely confirmed in hyperuricemic patients with stage III CKD, where 160 mg/day topiroxostat has been reported to decrease SUA levels and UAE [51].

Topiroxostat treatment was also shown to safely reach a significant decrease in SUA in hyperuricemic patients undergoing hemodialysis, at a lower dose rather than allopurinol [52]. Recently, the ETUDE trial has confirmed the positive impact of topiroxostat on renal function in patients with overt diabetic nephropathy [53]. However, clinical evidence on topiroxostat is yet limited.

## 3. Drugs That Inhibit the Reabsorption of Uric Acid: The Uricosurics

The uricosuric drugs increase the renal clearance of UA typically by inhibiting UA reabsorption in the renal proximal tubule.

To date, a putative role in UA secretion and reabsorption across the apical and the basolateral membrane of the proximal tubule has been assigned to several transporters [54] and genetic analyses such as genome-wide association studies [GWAS] have recently identified novel gene variants potentially associated with SUA levels and gout in humans [55]. The currently considered model involves a complex of proteins that serve the function of moving also other compounds across the proximal tubule’s epithelial cells, and that joining together form the “urate (or UA) transportasome”’ [56]. Among these transporter proteins, URAT1 (SLC22A12) plays a major role in the uptake of UA from the lumen [57]. Other transporters that are expressed in the apical membrane of kidney proximal tubule cells and with a role in the UA renal filtration are encoded by SLC17A1 (NPT1), SLC17A3 (NPT4), and ABCG2 (BCRP) [58].

GLUT9 (SLC2A9), located in the basolateral membrane, has a chief role in the transport of UA into the interstitium and blood [59]. A GWAS meta-analysis carried out on a sample of more than 28,000 individuals of European descent/White ethnicity has found SNPs associated with SUA levels in loci containing the SLC2A9, SLC17A1, SLC17A3, SLC22A12, and ABCG2 genes, among other novel loci [60]. A further GWAS meta-analysis has examined four phenotypes related to kidney function verifying that SLC2A9, SLC22A12, and ABCG2 loci are associated with UA concentration in 33,074 East Asian individuals [61]. A further GWAS meta-analysis including more than 140,000 European descents from the Global Urate Genetics Consortium [GUGC] has identified 16 novel and 10 previously known loci associated with SUA concentrations at genome-wide significance. These loci together explain up to 7% of the variance in SUA, with ABCG2 and SLC2A9 loci contributing to 3.4% of the total amount. 17 of the loci associated with urate concentrations were also associated with gout [62]. Many of the SNPs associated with SUA concentrations in the European analysis have also been associated in cohorts of Japanese (*n* = 15,286), African-Americans (*n* = 5820), and individuals of Indian ancestry (*n* = 8340), though there are some differences which may be correlated to the change in allele frequencies between the four different populations considered. Several other genes have a potential role in UA transport in the renal proximal tubule in humans. These all have in vitro evidence, and only in part have been reported in GWAS as associated with SUA concentrations and/or gout [63,64].

Uricosuric drugs can be safely administered in association with XOIs, offering promising perspectives for the treatment of refractory hyperuricemia [65]. Moreover, according to the EULAR [44] and ACR [45] recommendations for treating hyperuricemia, uricosurics are recommended as alternative first-line therapy for patients with failure to respond to XOIs or for those whose XOIs are contraindicated.

The main pharmacological characteristics of these drugs are resumed in Table 1.

### 3.1. Probenecid

Probenecid prevents the reabsorption of organic anions from the renal proximal tubule mainly by the inhibition of URAT1 (SLC22A12) transporter protein activity, as far as it is not excluded that it may also exert an effect upon OAT1 (SLC22A6), OAT4 (SLC22A11) and OAT10. Moreover, probenecid is a competitive inhibitor of the ATP release channel pannexin 1, being involved in the activation of the inflammasome, which releases IL-1ß that plays a pivotal role in the pathogenesis of atherosclerosis [66]. Contrarily, probenecid in vitro does not seem to affect GLUT9 (SLC2A9) at an effective pharmacological concentration of 1 mM [67].

Probenecid absorption is essentially complete following oral administration and its half-life in plasma ranged from 4 to 12 h, being dose-dependent. Its metabolism involves conjugation with glucuronic acid and oxidation of the alkyl side chains. Then, probenecid and its oxidized metabolites are largely bound to plasma proteins, especially to albumin [68]. Renal excretion is the major elimination pathway for the metabolites, while excretion of the parent drug is minimal and depends on urinary pH.

Probenecid can improve the allopurinol SUA-lowering effect, but this favorable interaction is reported to be significant only in patients with eGFR > 50 mL/min [27]. Contrarily, it should be used cautiously in subjects with CrCl < 50 mL/min, due to the lack of long-term safety and efficacy data. Likewise, the same caution should be used also in the treatment of the eldest patients [69]. However, a large observational study of 38,888 elderly gouty patients has recently associated probenecid treatment with a modesty decreased risk of cardiovascular events compared to allopurinol, including myocardial infarction, stroke, and heart failure exacerbation [70]. Anyway, probenecid treatment has not been demonstrated to affect endothelial function in middle-aged non-hypertensive, overweight individuals [71].

Finally, probenecid decreases renal elimination and, of consequence, augments the plasma concentration of some organic acids, such as antibiotics and NSAID indomethacin when used concomitantly [72].

In the United States, probenecid and colchicine are available in combination. This association may be suggested in patients who need SUA-lowering therapy having recurrent acute gout attacks [73].

### 3.2. Benzbromarone

Benzbromarone is a uricosuric drug that is considered more effective than probenecid. It inhibits UA transport by URAT1 (SLC22A12) and GLUT-9 (SLC2A9) in vitro, though its action upon GLUT-9 may be minimal at therapeutic doses [74]. In vitro, it also acts against UA uptake by OAT1 (SLC22A6) [75].

After oral administration, benzbromarone is quick but poorly absorbed in the upper digestive tract and reaches its peak plasma concentrations in around 2–4 h. Its plasma half-life ranges from 4 to 17 h [76].

Its liver metabolism allows benzbromarone administration in patients with mild to moderate CKD, where it seems to significantly delay the long-term development of end-stage renal disease most effectively than allopurinol [77], even if it should be avoided in patients with eGFR < 30 mL/min for the risk of hepatotoxicity [78]. Moreover, it is not indicated in patients with UA kidney stones or blood dyscrasias [75].

Because of its potential hepatotoxicity, benzbromarone has been withdrawn from the USA and some European markets. However, despite this concern, it is still commonly prescribed in several countries of South America and Asia [79].

### 3.3. Lesinurad

Lesinurad was conditionally approved in the United States at the end of 2015 and in the European Union in 2016. It is a selective UA reabsorption inhibitor targeting the URAT1 transporter [78]. In addition to URAT1, lesinurad also inhibits the OAT4 transporter, being involved in diuretic-induced hyperuricemia. However, unlikely probenecid, lesinurad does not inhibit OAT1 or OAT3: this may hypothetically result in fewer drug–drug interactions (e.g., with methotrexate and several antibiotics, antivirals, and antiretrovirals which interact with human OAT1-3 and whose concentrations can be increased by combining with probenecid) [80].

Lesinurad is a moderate inducer of the CYP3A cytochrome so that it may lead to reduced exposure of drugs metabolizes by this cytochrome, such as statins, sildenafil, amlodipine, indomethacine, and colchicines [81,82]. Furthermore, it might reduce the effectiveness of the hormonal contraceptives, so that additional contraception methods are strongly recommended in patients in treatment with lesinurad. Then, the lesinurad use is not recommended in patients just in treatment with inhibitors of epoxide hydrolase, such as the valproic acid.

A phase 3 clinical trial pooling data from gouty patients with a story of intolerance or contraindication to treatment with XOIs, found a high incidence of serum creatinine elevations and renal-related adverse events with lesinurad 400 mg monotherapy versus placebo [83]. For this reason, lesinurad should not be used as monotherapy. However, in combination with an XOI, lesinurad provides a dual mechanism to lower SUA by increasing renal UA excretion and reducing urate production. The CLEAR and the CRISTAL phase 3 trials respectively evaluated the effect of the addiction of lesinurad 200 or 400 mg or placebo to allopurinol ≥300 mg versus febuxostat 80 mg in monotherapy and combined with lesinurad 200 or 400 mg [84,85,86]. Safety data pooled from these phase 3 trials—overall including 1500 gouty patients—found a similar incidence of treatment-emergent adverse events across all the intervention groups.

A meta-analysis pooling data from 5 RCTs confirmed that lesinurad has an acceptable safety profile, with upper-respiratory-tract infection and hypertension occurring most commonly and transient renal-related events detected less frequently [87].

Data produced in the lesinurad dossier have led the European Medicines Agency [EMA] to contraindicate the administration of lesinurad monotherapy [88]. However, its use has been approved at a daily dose of 200 mg as an add-on therapy for patients who have had an inadequate response to the maximum tolerated doses of XOI, closely monitoring the renal function. In effect, lesinurad treatment has to be stopped if the serum creatinine levels increase more than two times the pre-treatment values or exceed 4.0 mg/dL. Finally, lesinurad is not recommended for patients with uncontrolled hypertension, unstable angina, recent myocardial infarction, or New York Heart Association [NYHA] class III or IV heart failure [89].

### 3.4. Arhalofenate

Arhalofenate inhibits the proximal tubular UA reabsorption by interacting with OAT4 and URAT2. Moreover, it reduces the urate-crystal stimulated release of IL-ß by modulating the peroxisome proliferator-activated receptor [PPAR] gamma pathway [90].

Arhalofenate is the first urate-lowering antiflare treatment, decreasing at a dosage of 800 mg gout flares compared with allopurinol 300 mg, without background colchicine, and with a similar incidence of treatment-emergent adverse events among the active treated groups [91]. In particular, arhalofenate might be relevant in the management of gout in the elderly, since they are often unable to tolerate long-term colchicine for flare prophylaxis and frequently have contraindications to corticosteroids and non-steroidal anti-inflammatory drugs [92]. However, arhalofenate safety and the efficacy have not just been tested in renal impairment. Moreover, allopurinol has been demonstrated to ensure a greater SUA reduction than arhalofenate, whose antiflare and uricosuric activities, of consequence, offer more promising perspectives in a combination regimen with an XOI [65]. In fact, a 12-week open-label phase 2 study has shown that 800 mg arhalofenate coadministered with 40 mg or 80 mg febuxostat exerts a more pronounced effect on SUA, with no reported deaths nor serious adverse events and no clinically relevant increase in serum creatinine [93].

Arhalofenate is currently in phase III clinical trials for the treatment of gout as add-on therapy to febuxostat. It had reached phase II/III clinical trials for the treatment of type 2 diabetes, but it was discontinued.

### 3.5. Verinurad

Verinurad is a high affinity and selective URAT1 inhibitor currently in development for the treatment of gout and asymptomatic hyperuricemia. This compound is one of the most potent URAT1 inhibitors yet identified and is highly specific for URAT1, with greater than 100-fold potency for URAT1 compared to the other transporters. In vivo, a single dose of verinurad 40 mg has demonstrated to lower SUA by up to 62% whereas multiple doses of verinurad 10 mg have reached a reduction in SUA levels approximately of 61%, being well tolerated at all doses and with no serious adverse events nor significant laboratory or ECG abnormalities reported [94]. Moreover, in coadministration with allopurinol 300 mg, verinurad (2.5–20 mg) has been shown to produce a dose-dependent decrease in SUA, with no instances of serum creatinine elevation [95].

The high potency of verinurad has favorably provided the opportunity to more completely define the molecular interaction of URAT1 with its inhibitors. A URAT1 binding assay using radiolabeled verinurad has revealed that distinct URAT1 inhibitors benzbromarone, sulfinpyrazone, and probenecid all inhibit verinurad binding via a competitive mechanism [96].

The efficacy and safety of verinurad has been recently evaluated in two phase II placebo-controlled clinical trials enrolling patients with gout or asymptomatic hyperuricemia. Even though verinurad monotherapy has resulted in sustained reductions in UA, several renal adverse events occurred [97].

### 3.6. Pegloticase

Pegloticase is a recombinant, pegylated mammalian uricase whose efficacy and safety have been tested in some clinical trials, where it has demonstrated to deeply lower serum urate, resolve tophi, reduce tender and swollen joint counts, decrease pain, and improve both patients’ global assessments and quality of life. Post hoc analyses of clinical results also indicated that chronic refractory gout patients not achieving sustained urate-lowering still have significant clinical benefits with pegloticase treatment [98]. Pegloticase also significantly decreases blood pressure in patients with chronic refractory gout but has no significant effect on renal function [99]. The major limitation of pegloticase is immunogenicity and the emergence of anti-drug antibodies that result in increased drug clearance, loss of efficacy, and infusion reactions, which can be prevented by the concomitant administration of immunosuppressant like methotrexate [100]. Furthermore, pegloticase is contraindicated in individuals with glucose-6-phosphate dehydrogenase (G6PD) deficiency [101]. For this reason, individuals at risk of this affection should undergo a prospective screening before starting treatment, to minimize the occurrence of pegloticase-related hemolytic anemia.

### 3.7. Other Uricosuric Drugs

There are drugs, which have not been specifically approved for use in gout, but with secondary uricosuric effects based on their pharmacodynamics. These drugs may be considered for treatment, especially in patients with comorbidities, which however would require these drugs.

The current assumption of hypouricemic effect following treatment with sodium-glucose co-transporter 2 [SGLT2] inhibitors is that glycosuria boosts excretion of UA in the urine. Actually, SUA reduction may be brought by a uricosuric effect secondary to glycosuria since hypouricemia and hyperuricosuria have been observed in subjects with familial renal glycosuria [102] hyperglycemic glycosuric type 1 diabetic patients, and euglycemic glycosuric type 1 diabetic patients following SGLT2 inhibition [103]. In vitro studies and clinical evidence have suggested that increased glucose concentration by gliflozins might carry out an increased efflux of UA into the lumen through GLUT9 in the proximal tubule [104].

The medications whose secondary effects might constitute a potential hyperuricemic treatment also include fenofibrate, a lipid-lowering drug activating intranuclear PPAR gamma whose metabolite fenofibric acid increases clearance of hypoxanthine and xanthine [105], and losartan, a unique angiotensin II receptor antagonist that inhibits renal tubular UA reabsorption increasing its urinary excretion. Both these drugs mainly target URAT1 [106,107]. Recently, the FIELD trial—which has been carried out on 9795 type 2 diabetic patients randomized to receive fenofibrate 200 mg/day or matching placebo for 5 years—has demonstrated that fenofibrate lowers SUA concentrations by up to 20% and almost halves first on-study gout events over the 5 years of treatment [108].

It is thought that also the ascorbic acid (vitamin C) might act as a uricosuric, inhibiting the UA reabsorption by URAT1 and playing a role in enhancing UA elimination [109]. In vitro, salicylate/salicyclic acid and indomethacin also inhibit the UA uptake by URAT1 [110]. By the way, a meta-analysis of 13 randomized controlled clinical studies have revealed that daily doses of 500 mg may reduce SUA levels and doses of >1000 mg/daily may even reduce the risk of developing gout [58].

## 4. Discussion

As well as several risk factors, including some medications, alcohol consumption, kidney disease, high blood pressure, hypothyroidism, obesity, are associated with an elevated risk of developing hyperuricemia, hyperuricemia seems to be an independent risk factor for the development of gout, but also coronary artery disease, CKD, type 2 diabetes mellitus, hypertension, atrial fibrillation and heart failure [111]. For this reason, optimizing SUA levels is increasingly a public health priority. In fact, SUA improvement could lead to a reduction in the risk of cardiovascular and renal events. In particular, a meta-analysis pooling data from 16 RCTs and including overall 1211 CKD patients, has recently shown that SUA-lowering therapy produces a 55% relative risk [RR] reduction (*p* < 0.001) for kidney failure events, and even a 60% RR reduction (*p* < 0.001) for cardiovascular events [112].

Today there is no clear evidence that lifestyle modification might be effective in decreasing SUA levels: insofar as a low-energy Mediterranean diet aimed at achieving optimal body weight seems reasonably the best approach [113]. Single nutrients and food have been associated with impaired or improved SUA levels, but definite data are yet lacking. However, in most of the cases, diet is not sufficient to optimize SUA level and a specific treatment is needed. The available urate-lowering drugs have very different efficacy and, even more, tolerability/safety profile. They can be easily grouped by mechanism of SUA decrease: XOIs reduce urate production, while uricosuric drugs (i.e., probenecid, benzbromarone, sulfinpyrazone, and lesinurad) improve SUA renal excretion by inhibiting its reabsorption. Another strategy for enhancing UA excretion consists in the addition of exogenous forms of urate oxidase which are not expressed in humans Urate oxidase breaks down UA to 5-hydroxyisourate, which is then spontaneously degraded to allantoin and more readily excreted through the kidneys because of its 5–10 folds greater solubility [114]. The final effect is obviously a significant reduction in UA [115].

Injectable uricases enzymatically degrade UA to allantoin despite a relevant SUA lowering effect [95,116,117]. Recombinant urate oxidases (rasburicase, pegloticase) are not good candidates for chronic hyperuricemia treatment, because of the high cost and a large number of side effects and contraindications [118].

The main pharmacological and clinical characteristics of UA-lowering drugs are resumed in Table 1.

In general, treatment persistence is a key factor for a positive clinical outcome throughout treatment for hyperuricemia. However, a large meta-analysis pooling data from 24 clinical studies showed that medication adherence to urate-lowering therapy among gout patients is poor, suggesting more attention is also needed from clinicians [120]. Moreover, considering the burden of cardiovascular risk connected to hyperuricemia, the use of lipid-lowering and antihypertensive drugs (e.g., losartan and fenofibrate) being able to improve SUA levels is highly recommendable, insofar as this approach has not just been clearly demonstrated to reduce the SUA-related risk in humans. This approach should be even more rigorous in patients with diabetes since the use of SGLT2 inhibitors rather than other hypoglycemic medications resulted in SUA reduction by up to –0.63 mg/dL [121]. Drugs impairing SUA levels (e.g., old-generation beta-blockers [122] and highly-dosed thiazides [123]) instead should be replaced with different antihypertensive drugs with neutral metabolic effects. Finally, considering the need for chronic treatment the choice of an SUA-lowering drug should be oriented by consideration about its safety and tolerability [124] other than the positive effect on kidney and cardiovascular outcomes.

Main clinical indications of the available serum uric acid lowering drugs are resumed in Table 2. Compared with other medical fields, the panel of SUA lowering drugs is supported by a relatively large and good quality evidence.

## 5. Conclusions

Xanthine oxidase inhibitors remain the safest and most effective SUA lowering drug for chronic treatment, while the efficacy of uricosuric agents is strongly modulated by pharmacogenetics. In particular, the 2020 ACR guidelines recommend the use of allopurinol as the preferred first-line urate-lowering therapy for all patients, including those with CKD, due to the respective cost and potential cardiovascular safety concerns recently emerged with febuxostat [125]. In patients with contraindication to allopurinol, febuxostat should be preferred to uricosuric drugs.

New emergent medications seem to be particularly effective in the acute management of refractory hyperuricemia, but their use is supported by a relatively small number of clinical trials. For this reason, further well-designed, long-term clinical trials are needed to deeper investigate their efficacy and safety profile.

## Figures and Tables

**Table 1:** Main pharmacological and clinical characteristics of the available serum uric acid lowering drugs.

	Drug	Oral Bioavailability	Protein Binding	Metabolism	Biological Half-Life	Degree of Uric Acid Reduction	Main Recorded Treatment-Emergent Adverse Events
Xantine Oxidase Inhibitors [15]	Allopurinol (C_5_H_4_N_4_O)	78 ± 20%	Negligible	Liver (80% oxipurinol, 10% allopurinol ribosides)	2–3 h (oxipurinol 14–30 h)	2.5% to 23.6%	Gastrointestinal distress, allopurinol hypersensitivity syndrome, skin rash, intestinal nephritis, hepatitis, eosinophilia
	Febuxostat (C_16_H_16_N_2_O_3_S)	>80%	99%	Liver (25–45% unchanged, 22–44% febuxostat acylglucuronide)	5–8 h	40% to 56%	Muscle pain, gastrointestinal disorders, elevation in liver enzymes
	Topiroxostat (C_13_H_8_N_6_)	80%	>92%	Liver (35.8% 2-hydroxy topiroxostat, 4.8% topiroxostat N-oxide, 43.3% N1-glucolonide, 16.1% gluculonide)	4.5–7.5 h	45.38 ± 21.8%	Elevation in liver enzymes, polyarthritis
Uricosurics [119]	Benzbromarone (C_17_H_12_Br_2_O_3_)	50%	>99%	Liver (6-hydroxybenzbromarone, 1′-hydroxybenzbromarone)	4–17 h	54% to 63%	Hepatotoxicity
	Probenecid (C_13_H_19_NO_4_S)	>99%	>90%	Liver (probenecid monoacyl glucuronide)	4–12 h	28.6% to 54%	Skin rash, gastrointestinal complaints, hypersensitivity
	Lesinurad (C_17_H_14_BrN_3_O_2_S)	>90%	>98%	65% Liver (Hydroxylated lesinurad, epoxide lesinurad), 35% Kidney (unchanged)	9–10 h	23% to 35%	Fatal cardiovascular events, nephrotoxicity,
	Arhalofenate (C_19_H_17_ClF_3_NO_4_)	>90	>90	Liver (unclear)	~50 h	12.5% to 16.5%	Increase in creatine kinase, kidney stones, angioedema, peripheral neuropathy
	Verinurad (C_20_H_16_N_2_O_2_S)	>90	>90	Liver (30% unchanged, 32% acylglucuronide M1 and 30% M8 n-oxide acyglucuronide 4)	~30 h	52% to 82%	Renal failure and increase in creatinine, gastrointestinal disorders
Drugs increasing SUA removal [100]	Peglotidase (C_1549_H_2430_N_408_O_448_S_8_)	Intravenous injection	0	Unclear	6.4–13.8 days	83%	Infusion-related reactions, vomiting, dyspnea, headache, urticaria, acute gout attacks, changes in blood pressure, methemoglobinemia

**Table 2:** Main clinical indications and characteristics of the available serum uric acid lowering drugs.

	Drug	Data Available from Large Clinical Trials (>10,000 Enrolled Participants Overall)	Current State of Drug Approval	Main Therapy	Add-on Therapy	Suitable for Use in Gout	Suitable for Use in Asymptomatic Hyperuricemic Subjects	Safely Administered in Renal Failure
Xantine-Oxidase inhibitors [15]	Allopurinol	Yes	Approved by EMA and FDA	Yes	No	No	Yes	No
	Febuxostat	Yes	Approved by EMA and FDA	Yes	No	Yes	No	Yes
	Topiroxostat	No	Approved by Japanese authorities only	Yes	No	Yes	Yes	Yes
Primarily uricosuric drugs [117]	Arhalofenate	No	Drug in development	No	Yes	Yes	No	NA
	Benzbromarone	No	Withdrawn for safety reasons	No	Yes	Yes	Yes	No
	Lesinurad	No	Conditionally approved by EMA and FDA	No	Yes	Yes	Yes	No
	Probenecid	Yes	Approved by EMA and FDA	Yes	Yes	Yes	Yes	No
	Verinurad	No	Not approved	No	Yes	Yes	Yes	NA
Other uricosuric drugs [107]	Fenofibrate	Yes	Approved by EMA and FDA °	No	Yes	NA	NA	No
	Losartan	Yes	Approved by EMA and FDA °	No	Yes	NA	NA	Yes
Urate oxidase [100]	Pegloticase	No	Approved by FDA though not by EMA	Yes	No	Yes	Yes	Yes

EMA = European Medicines Agency; FDA = Food and Drug Administration; NA = Not Available. ° Approved for indications different than hyperuricemia and/or gout.

## Data Availability

Not applicable.
